# Hysterectomy for Benign Gynecologic Disease: A Comparative Study of Articulating Laparoscopic Instruments and Robot-Assisted Surgery in Korea and Taiwan

**DOI:** 10.3390/medicina61081418

**Published:** 2025-08-05

**Authors:** Jun-Hyeong Seo, Young Eun Chung, Seongyun Lim, Chel Hun Choi, Tyan-Shin Yang, Yen-Ling Lai, Jung Chen, Kazuyoshi Kato, Yi-Liang Lee, Yu-Li Chen, Yoo-Young Lee

**Affiliations:** 1 Gynecologic Cancer Center, Department of Obstetrics and Gynecology, Samsung Medical Center, Sungkyunkwan University School of Medicine, Seoul 06351, Republic of Korea; 2 Department of Obstetrics and Gynecology, College of Medicine, National Taiwan University, Taipei 100233, Taiwan; 3 Department of Obstetrics and Gynecology, National Taiwan University Hospital Hsin-Chu Branch, Hsinchu 300195, Taiwan; 4 Department of Obstetrics and Gynecology, National Taiwan University Hospital Yun-Lin Branch, Yunlinounty 640203, Taiwan; 5 Department of Obstetrics and Gynecology, Kitasato University School of Medicine, Sagamihara 252-0329, Japan; 6 Department of Obstetrics and Gynecology, Tri-Service General Hospital National Defense Medical Center, Taipei 114202, Taiwan

**Keywords:** gynecologic surgery, articulating instruments, robotic surgery

## Abstract

*Background and Objectives*: Hysterectomy is a common non-obstetric procedure. Minimally invasive techniques, such as laparoscopy and robot-assisted surgery, have replaced open surgery for benign gynecologic conditions. Robotic surgery offers reduced blood loss and shorter hospital stays but is limited by high costs. Articulating laparoscopic instruments aim to replicate robotic dexterity cost-effectively. However, comparative data on these two approaches in hysterectomy are limited. *Materials and Methods*: This multicenter study analyzed the outcomes of hysterectomies for benign gynecological diseases using articulating laparoscopic instruments (prospectively recruited) and robot-assisted surgery (retrospectively reviewed). The surgeries were performed by minimally invasive gynecological surgeons in South Korea, Japan, and Taiwan. The baseline characteristics, operative details, and outcomes, including operative time, blood loss, complications, and hospital stay, were compared. Statistical significance was set at *p* < 0.05. *Results*: A total of 151 patients were analyzed, including 67 in the articulating laparoscopy group and 84 in the robot-assisted group. The operating times were comparable (114.9 vs. 119.9 min, *p* = 0.22). The articulating group primarily underwent dual-port surgery (79.1%), whereas the robot-assisted group required four or more ports in 71.4% of the cases (*p* < 0.001). Postoperative complications occurred in both groups, without a significant difference (9.0% vs. 3.6%, *p* = 0.17). No severe complications or significant differences in the 30-day readmission rates were observed. *Conclusions*: Articulating laparoscopic instruments provide outcomes comparable to robot-assisted surgery in hysterectomy while reducing the number of ports required. Further studies are needed to explore the learning curve and long-term impact on surgical outcomes.

## 1. Introduction

Hysterectomy remains one of the most commonly performed non-obstetric surgical procedures for women in the United States [1]. In recent decades, there has been a significant shift from open abdominal surgeries to minimally invasive techniques such as laparoscopy and robot-assisted surgery for the management of benign gynecological conditions [2,3]. Numerous studies have compared these minimally invasive approaches, revealing that, while robotic surgeries often have similar or even longer operative times, they are associated with reduced blood loss and shorter hospital stays than conventional laparoscopic procedures [4,5,6,7]. However, the high cost of robotic surgery remains a significant limitation, prompting the development of innovative instruments that aim to reduce costs, while offering functions comparable to those of robotic systems.

Devices such as articulating laparoscopic instruments have been introduced to address this issue. Articulating laparoscopic instruments offer improved dexterity and precision by replicating the wrist-like movements of robotic systems [8]. This innovation seeks to integrate the advantages of robotic surgery with laparoscopic procedures, thereby potentially improving surgical outcomes while maintaining cost-effectiveness [9]. Recent studies have demonstrated the feasibility of articulating instruments in various gynecological surgeries, including adnexal surgery, hysterectomy, and myomectomy [10,11,12]. These studies suggest that such instruments can enhance surgical performance and patient outcomes. However, to our knowledge, there is a lack of studies directly comparing the outcomes of hysterectomy performed with articulating laparoscopic instruments versus robot-assisted surgery for benign gynecologic diseases.

This study aimed to address this gap by evaluating and comparing the surgical outcomes of these two minimally invasive approaches for the treatment of benign gynecologic conditions.

## 2. Materials and Methods

This study compared the outcomes of hysterectomies using articulating laparoscopic instruments and robot-assisted surgery for benign gynecological diseases. This study was conducted between October 2022 and August 2024. Patients who underwent hysterectomy using articulating laparoscopic instruments were prospectively analyzed, whereas those who underwent robot-assisted surgery during the same period were retrospectively reviewed. Both surgical approaches were performed by expert gynecological surgeons specializing in minimally invasive surgeries in South Korea and Taiwan. At the start of the study, none of the participating surgeons had prior experience in articulating laparoscopic instruments. Each surgeon underwent at least 30 min of preoperative training with the instruments using a peg-transfer exercise, with additional training as required based on individual adaptation.

The study population included adult women aged 19 years or older who were scheduled for laparoscopic hysterectomy due to benign gynecologic conditions such as myoma, adenomyosis, and cervical dysplasia. Patients were excluded if malignancy was suspected preoperatively, necessitating lymphadenectomy or extra pelvic surgery. Informed consent was obtained from all patients who underwent surgery using articulating laparoscopic instruments.

The articulating laparoscopic instruments used in this study were ArtiSential^®^ devices (LIVSMED Inc., Seongnam-si, Republic of Korea), which feature 5 mm and 8 mm shafts with 360-degree articulation controlled by wrist and arm movements [8]. Depending on the surgeon’s preference and surgical requirements, various instrument types are used, including bipolar fenestrated forceps, bipolar Maryland dissectors, needle holders, monopolar scissors, and hooks. The articulating instrument was not used throughout the entire laparoscopic procedure but was selectively utilized during specific steps of the surgery, depending on the surgeon’s preference. All robotic surgeries were performed using the Da Vinci Xi system (Intuitive Surgical, Sunnyvale, CA, USA).

The baseline characteristics collected included age, body mass index (BMI), history and type of abdominal surgery, comorbidities, and American Society of Anesthesiologists (ASA) scores. The surgical details analyzed included whether adnexal surgery was performed, type of hysterectomy and adnexectomy, preoperative maximal uterine diameter (measured on ultrasound within six months prior to surgery), number of ports used, and types of articulating instruments employed.

The surgical outcomes evaluated in this study included the total operation time (defined as the time from skin incision to skin closure), conversion to laparotomy, presence of intraoperative adhesions, estimated blood loss (EBL), hemoglobin changes between preoperative levels and those measured 24 h postoperatively, weight of the uterus measured postoperatively, length of postoperative hospital stay, final pathological diagnosis, and postoperative complications. Adnexal surgeries performed alongside hysterectomies include salpingectomies in patients without adnexal diseases. The EBL was calculated by subtracting the volume of irrigating fluid from the total suctioned volume.

Statistical analyses were performed using SPSS version 28.0 (IBM SPSS Statistics, Armonk, NY, USA). Categorical variables were analyzed using the chi-square test or Fisher’s exact test, and continuous variables were assessed using Student’s *t* test or Mann–Whitney U test. Results are presented as means with standard errors (±SE) or medians with ranges. Statistical significance was set at *p* < 0.05.

This study was approved by the hospital’s institutional review board (IRB No. 2022-08-151). Prior to participation, patients were fully informed about the surgical procedures and the use of their data for clinical and research purposes, and they provided written informed consent.

## 3. Results

### 3.1. Patient Characteristics

A total of 151 patients were included in this study, of whom 67 underwent surgery using articulating laparoscopic instruments, and 84 underwent robot-assisted surgery. The demographic and clinical characteristics of the two groups are summarized in Table 1. Median age, BMI, comorbidities, and ASA scores were comparable between the groups. However, the history of at least one prior abdominal surgery was significantly more frequent in the robot-assisted group (46.4%) than in the articulating laparoscopic instrument group (32.8%).

### 3.2. Surgical Characteristics

As summarized in Table 2, the majority of patients in both groups underwent total hysterectomy, with no statistically significant difference observed in the type of hysterectomy performed (*p* = 0.11). Adnexal surgery was performed concomitantly with hysterectomy in both groups (*p* = 0.37), with salpingectomy being the most common procedure (64.2% in the articulating laparoscopic instrument group and 53.1% in the robot-assisted group, *p* = 0.27). The median maximum diameter of the uterus as measured preoperatively did not differ significantly between the groups (*p* = 0.53). A notable difference was observed in the number of ports used during surgery. In the articulating laparoscopic instrument group, 79.1% of the procedures were completed using two ports, whereas 71.4% of the procedures in the robot-assisted group required four or more ports (*p* < 0.001). Among the articulating instruments, scissors were the most frequently used (55.2%), followed by needle holders (26.9%), and hooks (16.4%).

### 3.3. Surgical Outcomes

Table 3 presents a comparison of surgical outcomes between the two groups. The total operation time was similar between the two groups, with a mean of 114.9 min for the articulating laparoscopic instrument group and 119.9 min for the robot-assisted group (*p* = 0.22). All surgeries were successfully completed laparoscopically without conversion to laparotomy. Despite the significant difference in the history of prior abdominal surgery, the incidence of intraoperative adhesions was comparable between the groups (*p* = 0.91). The median weight of the uterus removed was also similar between the groups, with both groups achieving successful removal of the uterus weighing up to 1000 g. The mean change in hemoglobin levels from preoperative to postoperative measurements was −1.6 g/dL in the articulating laparoscopic instrument group and −1.7 g/dL in the robot-assisted group (*p* = 0.32). Red blood cell transfusions were required in three patients in the articulating laparoscopic instrument group and two patients in the robot-assisted group due to blood loss. Postoperative pathological findings were consistent across the groups, with myomas being the most common diagnosis. Most patients were discharged on postoperative day two. Six patients in the articulating laparoscopic instrument group experienced postoperative complications compared to three in the robot-assisted group, but the difference was not statistically significant (*p* = 0.17). In the articulating laparoscopic instrument group, four patients experienced postoperative bleeding, necessitating red blood cell transfusions in two cases, although none required reoperation. Two patients were readmitted within 30 days of monitoring and treatment, and one additional patient required readmission owing to a wound infection. In the robot-assisted group, one patient experienced postoperative bleeding, and two patients had wound infections; however, no readmissions were reported. Although 30-day readmissions were more frequent in the articulating laparoscopic instrument group, the difference was not statistically significant (*p* = 0.05). No severe morbidities, defined as Clavien–Dindo classification (CDC) grade 3 or higher, were reported in either group.

## 4. Discussion

This study provides valuable insights into the comparative outcomes of hysterectomies performed using articulating laparoscopic instruments and robot-assisted surgery for benign gynecologic conditions. These findings suggest that articulating laparoscopic instruments can be a viable and cost-effective alternative to robotic surgery, with comparable surgical outcomes in terms of operative time, blood loss, and complication rates.

One notable observation was the higher prevalence of prior abdominal surgeries in the robot-assisted group (46.4%) than in the articulating laparoscopic instrument group (32.8%, *p* = 0.03). Despite this difference, the intraoperative adhesion rates were not significantly different between the groups (*p* = 0.91), indicating that a history of abdominal surgery did not substantially affect surgical outcomes. This finding highlights the feasibility of using both surgical approaches in patients with a history of abdominal surgeries. Regarding the uterine size, preoperative measurements were based on ultrasonography, which is inherently limited in terms of precision. However, the postoperative uterine weight did not differ significantly between the groups (median 345 g in the articulating laparoscopic instrument group vs. 368 g in the robot-assisted group, *p* = 0.09). This suggests that despite potential inaccuracies in preoperative ultrasound measurements, uterine size did not significantly affect the choice of surgical approach or outcome. Interestingly, the total operative time was comparable between the two groups (114.9 min vs. 119.9 min, *p* = 0.22). Although previous studies have shown some variability, there was no significant difference in operative times between robotic surgery and conventional laparoscopy during hysterectomy [6], and our findings are consistent with this. One experimental study demonstrated that ArtiSential^®^ instruments significantly improved dexterity among novice and intermediate users, with a learning plateau observed after several hours of peg-transfer training [13]. These results suggest that articulating instruments have a relatively short technical learning curve in simulated environments. However, in real-world clinical settings, surgeons may require additional operative experience to fully integrate these instruments into routine practice. Therefore, the comparable operative times observed in this study may, in part, reflect the early phase of the learning curve for articulating instrument use. As surgical familiarity increases, further reductions in operative time with articulating instruments may become evident. Considering surgical technique, a significant difference was observed in port usage. The articulating laparoscopic instrument group predominantly employed dual-port surgery (79.1%), whereas the robot-assisted group required four or more ports in 71.4% of the cases (*p* < 0.001). This reduction in the number of ports represents a distinctive advantage of articulating laparoscopic instruments. While patient-centered outcomes were not formally assessed in this study, it is reasonable to hypothesize that minimizing the number of incisions could contribute to improved cosmetic outcomes and potentially reduce postoperative discomfort. If these potential benefits are confirmed through future research, they would further support the role of articulating instruments as a less invasive alternative to robotic surgery. Prospective studies incorporating validated measures of scar satisfaction, pain scores, and quality of life are warranted to elucidate the broader impact of port reduction on patient recovery. Overall, our findings highlight the clinical utility of articulating instruments in laparoscopic hysterectomy, particularly in surgical settings where minimizing invasiveness is prioritized.

Postoperative complications were more frequent in the articulating laparoscopic instrument group, with six patients experiencing complications compared to three in the robot-assisted group. Additionally, three patients in the articulating instrument group required readmission within 30 days, whereas no readmissions were reported in the robot-assisted group. Although this difference did not reach statistical significance (*p* = 0.05), it may reflect the influence of unmeasured factors, such as institutional variations in postoperative care protocols or discharge criteria. It is also important to consider that articulating instruments were not employed throughout the entirety of the procedures; their use was predominantly limited to specific tasks such as cutting or suturing, rather than hemostasis. This selective application, coupled with the learning curve inherent to adopting new surgical technologies, may have contributed to the observed trend in postoperative morbidity. Moreover, previous studies have suggested that robot-assisted surgery may be associated with inherently lower complication rates than conventional laparoscopy, which could also account for part of the difference observed between the groups [6]. Future investigations should evaluate whether broader or more standardized integration of articulating instruments into laparoscopic workflows can enhance perioperative safety and reduce inter-institutional variability.

This study has several limitations that should be acknowledged. First, the retrospective nature of data collection for the robot-assisted surgery group may have introduced a degree of selection bias. To minimize this risk, both cohorts were drawn from the same observational period, and all procedures were performed by the same team of experienced minimally invasive surgeons, thereby ensuring consistency in surgical technique and operator expertise. Nonetheless, the possibility of residual bias cannot be entirely excluded. Additionally, the generalizability of our findings may be constrained by the exclusive use of a single articulating instrument system (ArtiSential^®^), which limits extrapolation to other platforms with differing ergonomics or performance characteristics. Furthermore, patient-reported outcomes were not evaluated in this study. These include important clinical parameters such as postoperative pain, cosmetic satisfaction, and overall quality of life, which are particularly relevant in the context of minimally invasive surgery. The analysis was also limited to short-term outcomes, and long-term complications such as adhesion formation, chronic pelvic pain, or reoperation rates were not assessed. All surgeries were conducted at high-volume tertiary centers in Korea and Taiwan, which may limit the applicability of our findings to other healthcare settings. Lastly, the use of articulating instruments was not standardized across cases; they were selectively applied according to the surgeon’s discretion, introducing potential heterogeneity in exposure and effect.

Future research should aim to address these limitations through prospective, multicenter trials that incorporate standardized protocols for articulating instrument use. Comparative studies involving multiple device platforms and diverse healthcare environments are warranted to enhance the generalizability of findings. Moreover, the integration of validated patient-reported outcome measures and long-term follow-up will be essential to comprehensively evaluate the clinical value and patient-centered benefits of articulating laparoscopic technology.

## 5. Conclusions

In conclusion, this study demonstrated that articulating laparoscopic instruments offer comparable surgical outcomes to robot-assisted surgery in the treatment of benign gynecologic conditions, particularly in performing hysterectomy. Future prospective randomized studies are needed to confirm these findings and optimize the integration of articulating instruments into routine clinical practice.

## Figures and Tables

**Table 1:** Baseline characteristics of patients.^1^

	Laparoscopy with ArtiSential^®^ (*n* = 67)	Robot-Assisted Surgery (*n* = 84)	*p* Value
Age (y)	47 (28–79)	48 (31–75)	0.82
BMI (kg/m^2^)	23.5 (19.2–42.0)	23.1 (17.7–38.0)	0.58
No. prior abdominal surgeries			0.03
None	45 (67.2)	45 (53.6)	
1	21 (31.3)	31 (36.9)	
2	1 (1.5)	7 (8.3)	
≥3	0 (0.0)	1 (1.2)	
Type of abdominal surgery			<0.001
Cesarean section	5 (7.5)	29 (34.5)	
Adnexa surgery	6 (9.0)	1 (1.2)	
Myomectomy	5 (7.5)	5 (6.0)	
Other gynecologic surgeries	2 (3.0)	5 (6.0)	
Appendectomy	3 (4.5)	0 (0.0)	
Others	2 (3.0)	6 (7.1)	
Comorbidity			0.24
Hypertension	4 (6.0)	11 (13.1)	
Diabetes mellitus	3 (4.5)	5 (6.0)	
Dyslipidemia	5 (7.5)	4 (4.8)	
Thyroid disease	11 (16.4)	8 (9.5)	
Others	21 (31.3)	13 (15.5)	
ASA score			0.45
I, II	65 (97.0)	82 (97.6)	
III	1 (1.5)	2 (2.4)	
IV	1 (1.5)	0 (0.0)	

BMI, body mass index; ASA, American Society of Anesthesiologists. ^1^ Data are presented as median (range) or no. (%).

**Table 2:** Surgical characteristics.^1^

	Laparoscopy with ArtiSential^®^ (*n* = 67)	Robot-Assisted Surgery (*n* = 84)	*p* Value
Type of operation			0.37
Hysterectomy with adnexa surgery	67 (100.0)	83 (98.8)	
Hysterectomy without adnexa surgery	0 (0.0)	1 (1.2)	
Type of hysterectomy			0.11
Total hysterectomy	65 (97.0)	84 (100.0)	
Subtotal hysterectomy	2 (3.0)	0 (0.0)	
Type of adnexa surgery			0.27
Salpingo-oophorectomy	15 (22.4)	27 (32.5)	
Cystectomy	9 (13.4)	2 (2.4)	
Cystectomy + salpingectomy	0 (0.0)	10 (12.0)	
Salpingectomy	43 (64.2)	44 (53.1)	
Preoperative maximal diameter of uterus (mm)	103 (44–115)	94 (45–190)	0.53
Number of ports			<0.001
1	0 (0.0)	11 (13.1)	
2	53 (79.1)	3 (3.6)	
3	2 (3.0)	10 (11.9)	
≥4	12 (17.9)	60 (71.4)	
Type of articulating instrument			N/A
Dissector	1 (1.5)	-	
Scissors	37 (55.2)	-	
Hook	11 (16.4)	-	
Needle holders	18 (26.9)	-	

^1^ Data are presented as median (range) or no. (%).

**Table 3:** Operative and postoperative outcomes.^1^

	Laparoscopy with ArtiSential^®^ (*n* = 67)	Robot-Assisted Surgery (*n* = 84)	*p* Value
Total operation time (minutes)	114.9 ± 22.9	119.9 ± 26.5	0.22
Conversion to laparotomy			1.0
No	67 (100.0)	84 (100.0)	
Yes	0 (0.0)	0 (0.0)	
Intraoperative adhesion			0.91
No	57 (85.1)	72 (85.7)	
Yes	10 (14.9)	12 (14.3)	
EBL (mL)	100 (10–650)	100 (20–400)	0.18
Hemoglobin change ^2^ (g/dL)	−1.6 ± 1.2	−1.7 ± 1.1	0.32
Weight of uterus (g)	345 (33–961)	368 (55–1000)	0.09
Transfusion of RBC			0.48
No	64 (95.5)	82 (97.6)	
Yes	3 (4.5)	2 (2.4)	
Number of postoperative hospital stays (days)			0.54
1	1 (1.5)	1 (1.2)	
2	59 (88.1)	77 (91.7)	
3	5 (7.5)	5 (6.0)	
4	1 (1.5)	0 (0.0)	
≥5	1 (1.5)	1 (1.2)	
Pathologic diagnosis			0.57
Myoma	24 (35.8)	38 (45.2)	
Adenomyosis	19 (28.4)	12 (14.3)	
Cervical dysplasia	2 (3.0)	5 (6.0)	
Endometrial hyperplasia	3 (4.5)	7 (8.3)	
Others	19 (28.4)	22 (26.2)	
Postoperative complication			0.17
No	61 (91.0)	82 (96.4)	
Yes	6 (9.0)	3 (3.6)	
Details of complication			0.15
Postoperative bleeding	4 (6.0)	1 (1.2)	
Wound infection	1 (1.5)	2 (2.4)	
Urinary tract infection	1 (1.5)	0 (0.0)	
Readmission within 30 days			0.05
No	64 (95.5)	84 (100.0)	
Yes	3 (4.5)	0 (0.0)	
Morbidity (CDC grade III–IV)	0	0	N/A

EBL, estimated blood loss; RBC, red blood cell; CDC, Clavien–Dindo classification; SD, standard deviation. ^1^ Data are presented as median (range), mean ± SD or no. (%). ^2^ Hemoglobin change refers to postoperative hemoglobin minus preoperative hemoglobin.

## Data Availability

The data that support the findings of this study are available from the corresponding author upon reasonable request.

## Abbreviations

The following abbreviations are used in this manuscript:

BMI	Body mass index
ASA	American Society of Anesthesiologists
ELB	Estimated blood loss
SE	Standard errors
CDC	Clavien–Dindo classification
SD	Standard deviation
RBC	Red blood cell
